# A Case of Acute Aortic Dissection Complicated by Bowel Malperfusion

**DOI:** 10.7759/cureus.67117

**Published:** 2024-08-18

**Authors:** Jing Huang, Siya Khanna, Max Macbarb

**Affiliations:** 1 Medicine, Nova Southeastern University Dr. Kiran C. Patel College of Osteopathic Medicine, Davie, USA; 2 Osteopathic Medicine, Nova Southeastern University Dr. Kiran C. Patel College of Osteopathic Medicine, Fort Lauderdale, USA; 3 Emergency Medicine, Stony Brook Southampton, Southampton, USA

**Keywords:** malperfusion, hypertension, cocaine use, bowel ischemia, acute aortic dissection

## Abstract

Acute aortic dissection (AAD) is a life-threatening condition with high mortality rates. Prompt diagnosis and intervention are crucial to minimize complications; high suspicion for AAD should be exercised in patients presenting with acute sheering chest pain. While obtaining a proper history and performing a physical examination are important in the diagnostic process, not all cases present with typical symptoms. This can make diagnosis challenging, especially in patients who present with cognitive disorientation, making it difficult to take a proper history.

We present the case of a 57-year-old male who presented to the emergency department (ED) of a community hospital with cognitive disorientation and abdominal pain that began two days prior to presentation and was associated with nausea, vomiting, and diarrhea. Laboratory results showed that the patient had an elevated white blood cell count, hyperkalemia, acute renal injury, and elevated lactate levels. Initial chest radiography and computed tomography showed no acute findings. Urine toxicology was positive for cocaine. The elevated lactate levels and cocaine use prompted us to order a computed tomography angiography (CTA) of the abdomen and pelvis with suspicion for bowel ischemia. He was found to have an abdominal aortic dissection with extrinsic compression of the patent true lumen. He was then transferred to a tertiary care facility, and a repeat CTA of the chest, abdomen, and pelvis showed a type A aortic dissection with extensive bowel ischemia. The patient was deemed too unstable for surgical repair and expired. The case highlights the challenges of diagnosing AAD due to its varied presentations and emphasizes the importance of maintaining a high suspicion for the condition in high-risk individuals. Additionally, the case highlights the potential complications associated with AAD.

## Introduction

Acute aortic dissections (AADs) represent a critical cardiovascular emergency, with mortality rates estimated at 40% upon presentation [1]. Prompt identification and evaluation are crucial for AAD, as it can rapidly trigger life-threatening complications in the cardiovascular, neurological, and gastrointestinal systems due to compromised blood flow. Treatment strategies depend on the specific anatomy involved, with imaging and classification systems guiding management [2]. Despite its relative infrequency (5-30 cases per million annually, with a peak between 50 and 65 years of age) [2], AAD diagnosis in the emergency department (ED) is often delayed due to its varied presentations and the presence of other critical conditions. Patients may exhibit diverse symptoms such as shearing chest pain, abdominal/back pain, weakness, shortness of breath, or signs of shock. The aortic wall is comprised of three layers called the intima, media, and adventitia [2]. The pathophysiology of an aortic dissection (AD) occurs when high pressures cause stress on the aortic walls, leading to tears in the intimal layer and causing blood to pool into the medial space, creating a false lumen [1,2]. Considering that the pathophysiology of a dissection is caused by high pressure and/or weakened walls, it is unsurprising that risk factors for AAD include uncontrolled hypertension (HTN), history of aortic aneurysm, sympathomimetic drug use, or genetic conditions such as Marfan syndrome [2]. There should be a heightened suspicion for individuals with a history of aortic disease or relevant lifestyle factors that present to the ED with chest pain. In this case report, we present a case highlighting the diagnostic challenges of AAD, particularly when presenting with atypical symptoms. Our patient initially presented with non-specific abdominal pain, ultimately diagnosed with AAD complicated by bowel ischemia. Through this scenario, we delve into the intricacies of healthcare delivery, emphasizing the challenges and considerations encountered in diagnosing and managing AAD.

## Case presentation

A 57-year-old male presented to the ED of a community hospital complaining of severe left-sided abdominal pain rated 8/10 that began two days prior to presentation. The pain was continuous and accompanied by nausea, vomiting, and diarrhea. The patient suspected a viral infection as the cause and admitted to taking benzodiazepines the night before to aid sleep. His symptoms worsened, which prompted his presentation to the ED. His medical history included HTN and marijuana use. Obtaining additional history was challenging due to cognitive disorientation.

**Figure 1:**
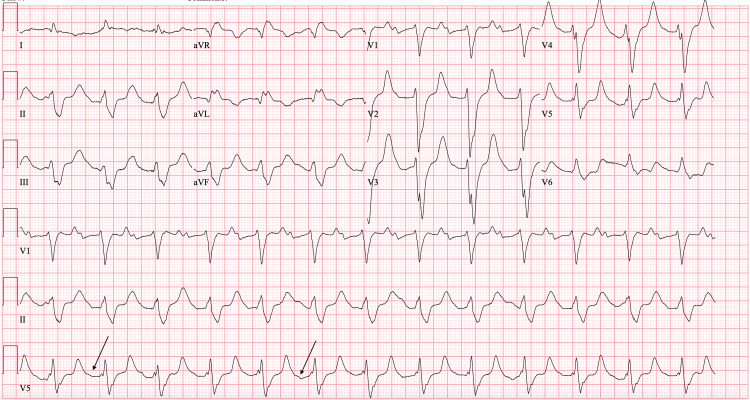
Electrocardiogram showing first-degree atrioventricular block (black arrows) with nonspecific ST segment/T wave abnormalities.

**Figure 2:**
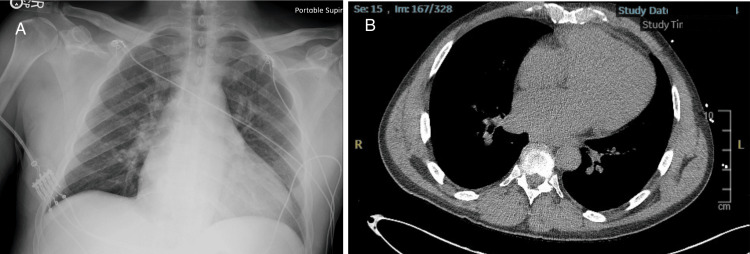
Initial chest radiography (A) and computed tomography of the chest without contrast (B) indicating mild cardiomegaly with no acute findings.

**Figure 3:**
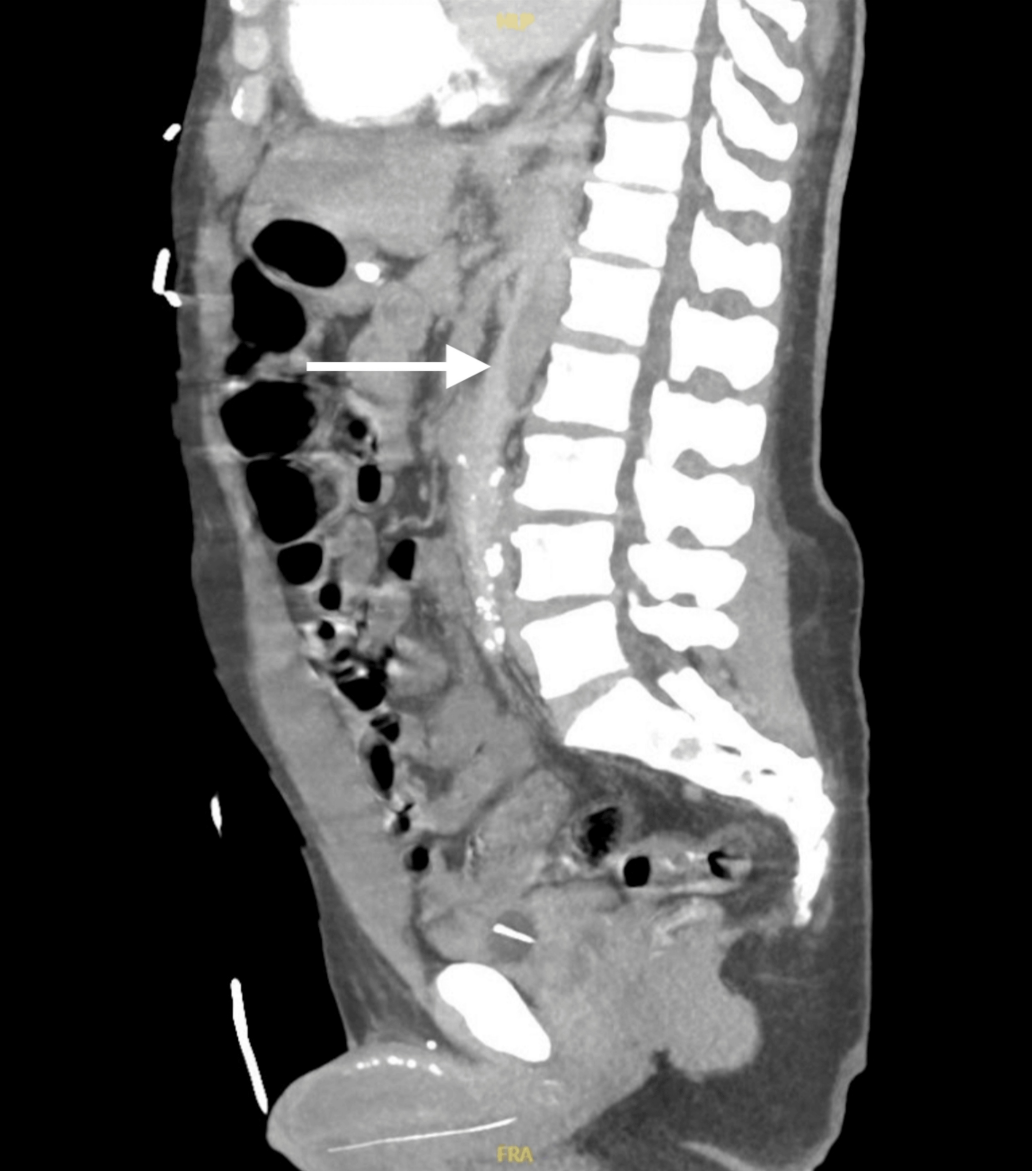
A sagittal section of a CT angiogram of the abdomen and pelvis showing an abdominal aortic dissection with extrinsic compression of the patent true lumen, as indicated by the arrows.

**Figure 4:**
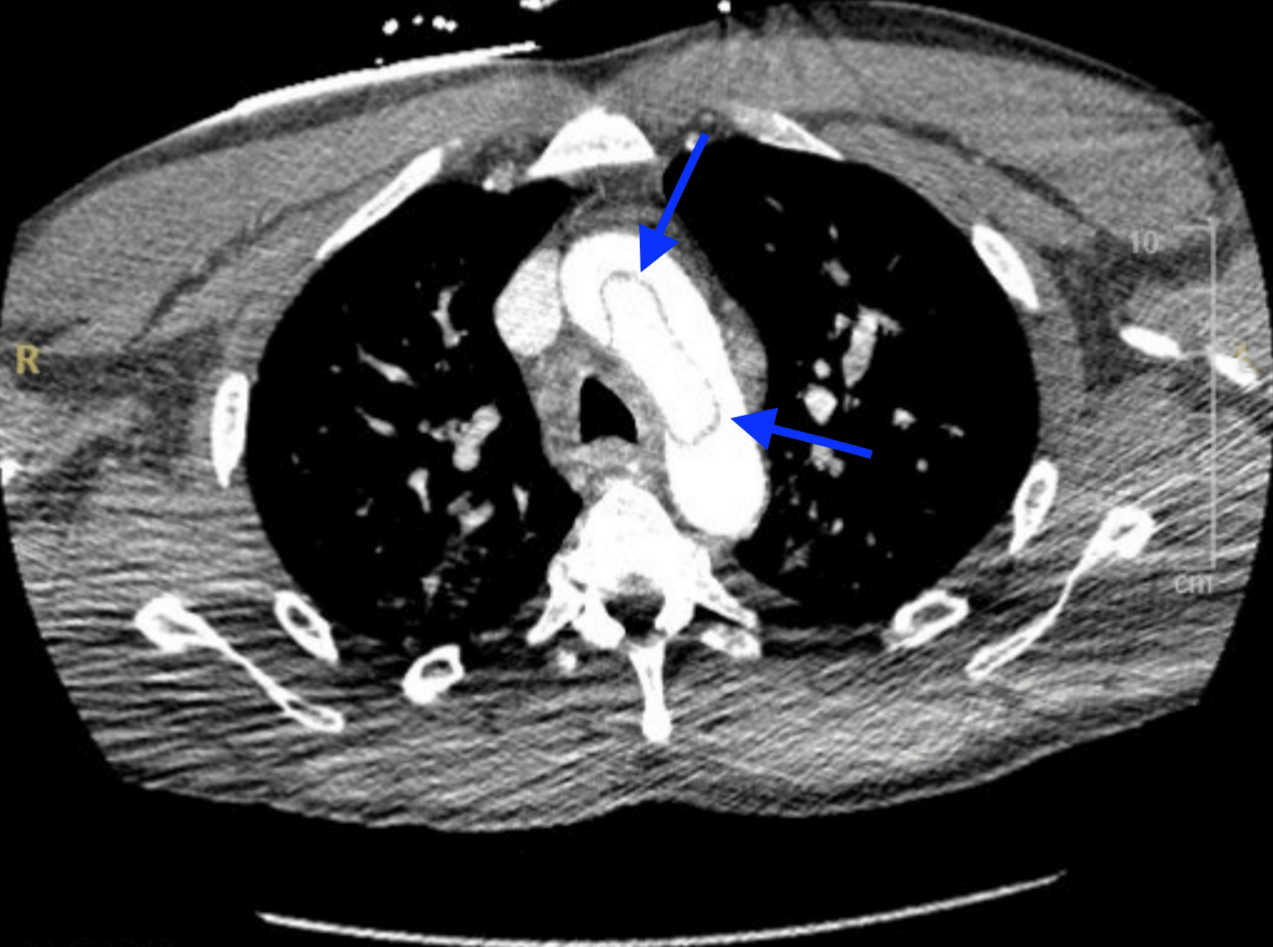
Transverse section of CT angiography of the chest showing an aortic dissection involving the aortic arch, as indicated by the blue arrows.

**Figure 5:**
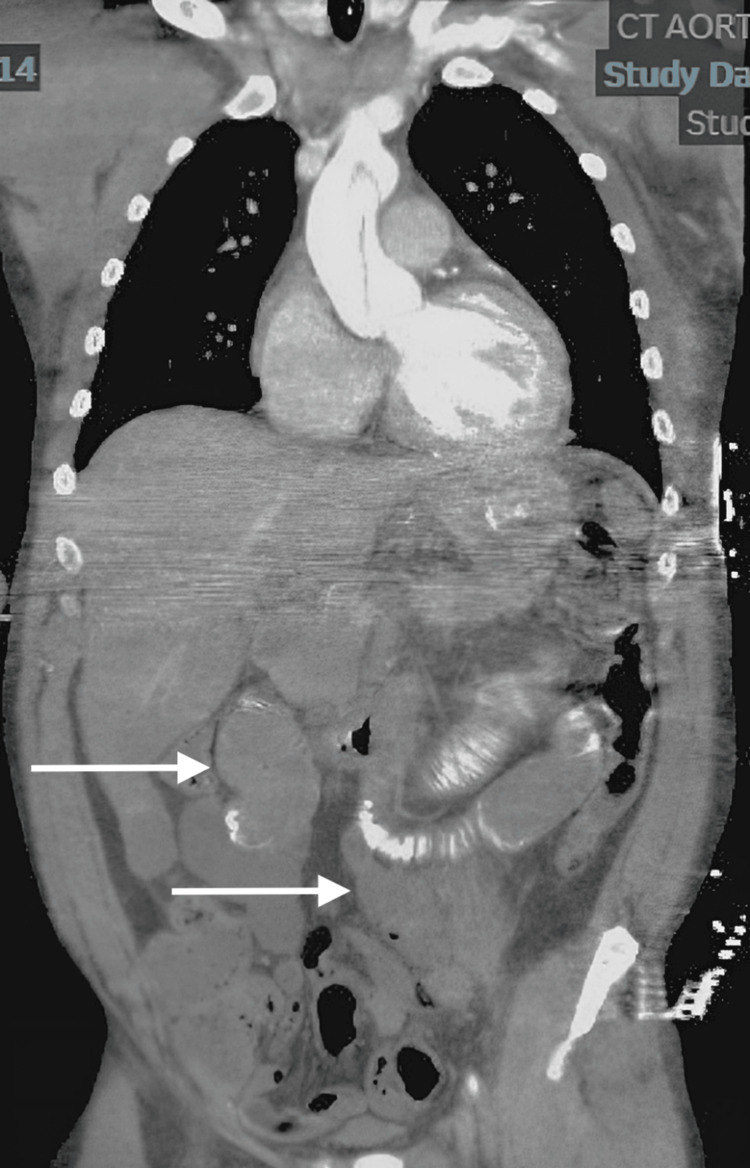
Frontal section with white arrows indicating areas lacking mucosal enhancement in the small bowel and colon consistent with mesenteric ischemia.

In the ED, initial vital signs revealed a blood pressure of 96/45 mmHg, heart rate of 61 beats per minute, respiratory rate of 32 breaths per minute, oxygen saturation of 94% on room air, and temperature of 97.8°F. The patient was noted to be in moderate distress. On physical examination, the patient exhibited tenderness to palpation on the left side of the abdomen with guarding. Faint pulses were also noted in both upper and lower extremities. Although the patient was tachypneic, breath sounds were normal bilaterally. The neurological examination revealed moderate altered mental status, while the cardiac examination was unremarkable. An electrocardiogram (ECG) showed first-degree atrioventricular block with nonspecific ST segment/T wave abnormalities (Figure 1). Laboratory results showed white blood cell count 15.74 x10^3^/μL, hemoglobin 15.4 g/dL, hematocrit 46.8%, platelet count 319 x10^3^/μL, potassium 9 mmol/L, glucose 46 mg/dL, blood urea nitrogen 65 mg/dL, creatinine 6.67 mg/dL, alanine aminotransferase 1,546 U/L, aspartate aminotransferase 3361 U/L, and lactic acid 9.6 mmol/L. Chest X-ray (Figure 2a) and computed tomography (CT) of the chest, abdomen, and pelvis without contrast (Figure 2b) revealed mild cardiomegaly with no acute findings. Urine toxicology was positive for cocaine and benzodiazepine. One liter of normal saline was administered, and initial treatment for hyperkalemia was begun with plans for emergent dialysis. The patient's condition continued to deteriorate with a repeated lactic acid level of 10.7 mmol/L. Given the elevated lactate levels, a CTA of the abdomen and pelvis was performed to rule out mesenteric ischemia. Imaging revealed an abdominal AD with extrinsic compression of the patent true lumen (Figure 3). The flap was noted at the level of the celiac and superior mesenteric arteries. Consequently, a decision was made to transfer the patient to a tertiary care center. At the time of transfer, the patient's blood pressure was 57/42 mmHg, with a mean arterial pressure of 47 mmHg. Pressors were withheld to allow for permissible hypotension and contain the dissection. Upon arrival at the tertiary care center, a repeat CTA of the chest, abdomen, and pelvis confirmed a type A AD involving the aortic arch (Figure 4) with extensive bowel ischemia (Figure 5). Unfortunately, the patient was deemed too unstable for surgery and subsequently passed away.

## Discussion

The clinical manifestation of AD poses a diagnostic challenge, given its diverse array of symptoms that may overlap with other acute medical conditions. While severe chest pain radiating to the back is a hallmark, not all patients exhibit this classic presentation [2]. Atypical symptoms such as syncope, dyspnea, or abdominal pain can obscure diagnosis, leading to delayed treatment [3]. The location and extent of the dissection further complicate matters, influencing symptom patterns; for instance, ascending aortic involvement may mimic acute coronary syndromes, while descending aortic involvement may cause back or abdominal pain [4]. As highlighted earlier, AD results from a complex interplay of various predisposing factors. While HTN remains the predominant primary factor, lifestyle choices such as smoking and illicit drug use, advancing age, male gender, and a familial history of aortic disease augment the risk [2]. In this patient's scenario, multiple risk factors predisposed him to AD including age, HTN, and cocaine use. Cocaine serves as a potent vasoconstrictor, triggering a surge in catecholamines that physiologically elevate blood pressure and heart rate [5]. This sudden elevation can precipitate intimal tearing or exacerbate an existing dissection. Cocaine use causes premature atherosclerosis in the distal ascending aorta and is often associated with chronic, uncontrolled HTN, which may contribute to the increased likelihood of type B AD [6]. Presenting patients also tend to be younger [6]. Coupled with his history of HTN, cocaine use represented an additional risk factor, likely exacerbating the expansion of his AD.

The diagnosis of AAD can be achieved through various imaging modalities such as CTA, magnetic resonance imaging (MRI), or transesophageal echocardiogram (TEE) [7]. TEE is useful in unstable patients as it can be performed at bedside, but this technique requires esophageal intubation, making it an invasive procedure with a risk of esophageal perforation [7]. An MRI has less practical uses in an acute setting due to its cost and time needed to obtain the images. In a stable patient, CTA is the gold standard for diagnosing AD because of its availability, accessibility, and diagnostic accuracy [7]. Other imaging modalities, such as chest X-rays used for initial screening, can assist in diagnosing AD. However, the detection of a widened mediastinum on chest X-rays, which is indicative of AD, has declined in recent years. This decline is likely due to several factors, including the reduced use of chest X-rays compared to chest CT scans for diagnosing AD [3]. The sensitivity for detecting an AD via chest X-ray is also relatively low at 70%; hence, a normal chest X-ray should not be used to rule out an AD [8]. Imaging plays a crucial role in classifying the type of dissection, which, in turn, determines the appropriate management strategy. Stanford type A dissections involving the ascending aorta necessitate surgical emergency due to the potential for acute tamponade or aortic regurgitation, whereas type B dissections, which are distal and do not involve the ascending aorta, are typically managed medically by stabilizing blood pressure and heart rate [9,10].

Concurrent complications that can lead to increased mortality and poor prognosis in patients with AAD include organ malperfusion and unstable hemodynamics [1,2]. Organ malperfusion occurs in approximately 30% of AD cases and presents with symptoms such as abdominal pain, abnormal lactate levels, pulseless lower extremity, and renal/liver dysfunction [11]. Common malperfused organs include cerebral, coronary, limb, renal, and mesenteric, with mesenteric making up 2.4% of cases [11]. In our patient, who exhibited abdominal pain and elevated lactate levels, imaging confirmed our suspicion of mesenteric ischemia. Regrettably, owing to the extensive nature of the ischemia, surgical intervention was deemed unfeasible and the patient ultimately expired. Numerous factors compounded the heightened complications and intricacies of this patient's case, including delayed presentation to seek medical care, necessitating a hospital transfer to a tertiary care center, and his concurrent cocaine use.

## Conclusions

AAD is a time-sensitive emergency with a high mortality rate if not diagnosed and treated promptly. Timely recognition is essential, as delayed diagnosis can lead to devastating complications such as aortic rupture and death. Clinicians should maintain a high index of suspicion for AAD, especially in high-risk individuals and those presenting with atypical symptoms. In-depth exploration of lifestyle factors, including sympathomimetic use, should be included in the evaluation of patients with potential AAD. This case, where the patient presented with cognitive disorientation and abdominal pain, highlights the challenges of diagnosing AAD with atypical presentations. It emphasizes the importance of considering AAD in patients with a broad range of symptoms, even in the absence of classic signs such as chest pain. Additionally, the presence of cognitive disorientation may hinder history taking, making urine toxicology screening valuable when suspicion for AAD is high.
